# Glia-related circadian plasticity in the visual system of Diptera

**DOI:** 10.3389/fphys.2013.00036

**Published:** 2013-08-23

**Authors:** Jolanta Górska-Andrzejak

**Affiliations:** Department of Cell Biology and Imaging, Institute of Zoology, Jagiellonian UniversityKraków, Poland

**Keywords:** circadian plasticity, glia, visual system, *Drosophila*, *Musca*

## Abstract

The circadian changes in morphology of the first visual neuropil or lamina of Diptera represent an example of the neuronal plasticity controlled by the circadian clock (circadian plasticity). It is observed in terminals of the compound eye photoreceptor cells, the peripheral oscillators expressing the clock genes. However, it has been found also in their postsynaptic partners, the L1 and L2 monopolar cells, in which the activity of the clock genes have not yet been detected. The circadian input that the L1 and L2 receive seems to originate not only from the retina photoreceptors and from the circadian pacemaker neurons located in the brain, but also from the glial cells that express the clock genes and thus contain circadian oscillators. This paper summarizes the morphological and biochemical rhythms in glia of the optic lobe, shows how they contribute to circadian plasticity, and discusses how glial clocks may modulate circadian rhythms in the lamina.

## Introduction

The visual system of Diptera exhibits daily rhythmic changes in morphology and physiology (Pyza and Górska-Andrzejak, 2008; Pyza, 2010). To maintain synchrony with environmental cycles, this rhythmicity is entrained by an external cycle of day and night (LD), but it is generated predominantly by the endogenous pacemakers, the circadian clocks, that produce oscillations with a period of approximately 24 h. Due to timing signals from the clocks, the phenomenon of rhythmic changes persists also in the condition of constant darkness (DD), representing an example of a particular type of plasticity, the circadian plasticity (Frenkel and Ceriani, 2011; Mehnert and Cantera, 2011).

## Circadian plasticity of neurons in the visual system of Diptera

**Figure 1:**
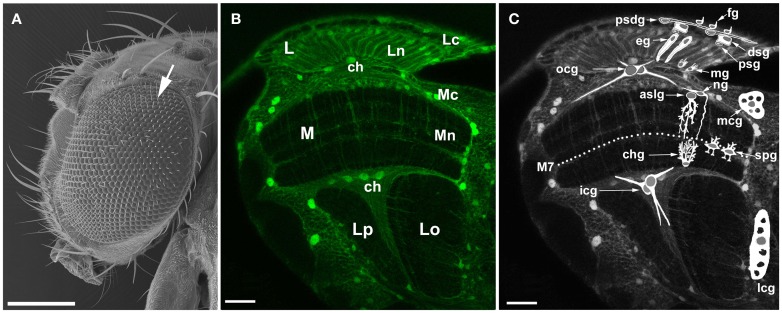
**The visual system of the fruit fly, *Drosophila melanogaster*. (A)** Scanning electron micrograph of a head and a large compound eye. The eye is composed of approximately 800 hexagonal units called facets or ommatidia (arrow). The ommatidial array of photoreceptors in the retina receives photic and visual information, transduces it into receptor action potentials and transmits to underlying optic lobe. Scale bar: 200 μm. **(B)** Confocal image of the optic lobe of transgenic flies Repo-Gal4 × UAS-S65T-GFP, in horizontal section. Targeted expression of Green Fluorescence Protein (GFP) to glial cells reveals the general morphology of the optic lobe. There are three synaptic regions (neuropils) beneath the retina of the compound eye: the lamina (L), the medulla (M), and the lobula that in Diptera consists of the lobula (Lo) and the lobula plate (Lp). Lc, lamina cortex; Ln, lamina neuropil; Mc, medulla cortex; Mn, medulla neuropil; ch, chiasm. Scale bar: 20 μm. **(C)** Schematic representation of so far identified types of glia (based on Edwards et al., 2012) revealing their general morphology and relative locations in the optic lobe: fg, fenestrated glia; psdg, pseudocartridge glia; dsg, distal satellite glia; psg, proximal satellite glia; eg, epithelial glia; mg, marginal glia; mcg, medulla cortex glia; aslg, astrocyte-like glia of the distal medulla neuropil; ng, another type of the distal medulla neuropil glia; spg, serpentine glia; chg, chandelier glia; ocg, outer chiasm glia (giant and small ocg); icg, inner chiasm glia; lcg, lobula cortex glia; M7, the serpentine layer. Certain types of glia (eg, aslg, and/or ng, mcg, lcg, ocg, icg) can be also discern in the tissue visible in the background being marked by GFP. Scale bar: 20 μm.

**Figure 2:**
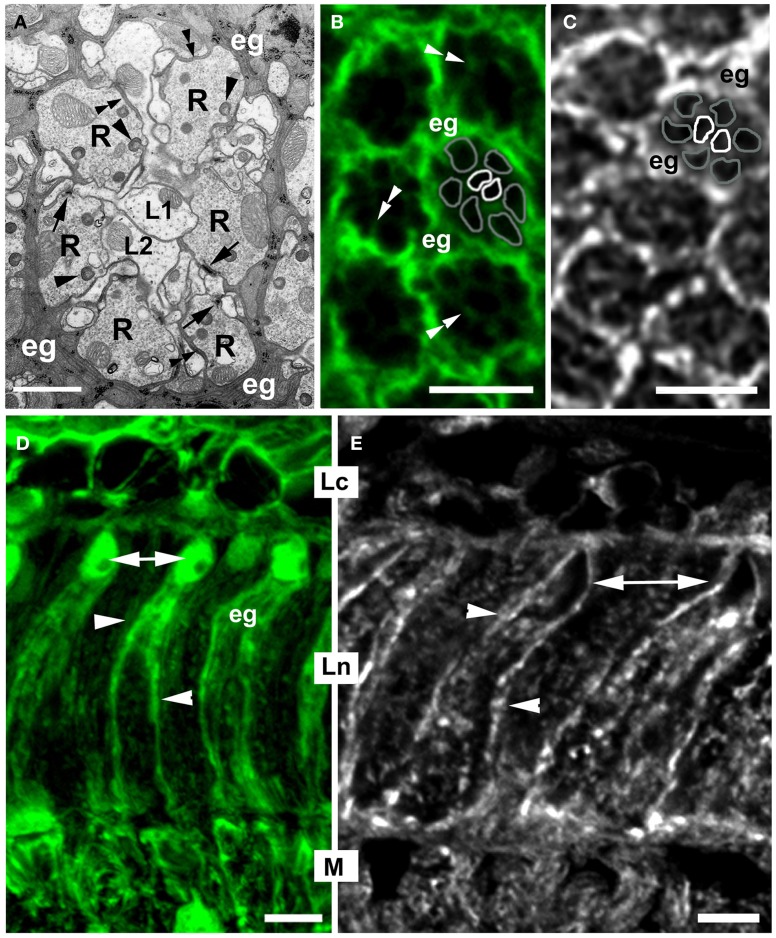
**The morphology of the cartridge in cross- (A–C), and longitudinal (D,E) section of *Drosophila melanogaster* first visual neuropil or lamina. (A)** EM microphotograph of a single cartridge. The cylindrical terminals of six photoreceptors (R) surround axons of their main target cells, the L1 and L2 lamina interneurons, the so called large monopolar cells (LMCs). Apart from these, each cartridge hosts also smaller processes (not marked) of two more photoreceptors, R7 and R8, and three other monopolar cells, L3–L5, as well as profiles of amacrine cells, whose cell bodies are located in the second visual neuropil or medulla, and tangential neurons with cell bodies in other parts of the brain. Each cartridge is enwrapped by three cells of epithelial glia (eg) that send small processes into the cartridge (double arrowheads) and deep, dynamic invaginations with a spherical head, called capitate projections (arrowheads) into photoreceptor terminals. R-terminals contain profiles of presynaptic ribbons (T-bars) of the tetrad synapses (arrows), the most abundant type of synapses in the lamina. They constitute approximately 59% of the total number of all synapses of a cartridge (Meinertzhagen and Sorra, 2001). These synapses transmit photic and visual information received by ommatidia toward the brain. Scale bar: 1 μm. **(B)** Confocal image of the cartridges of Repo-Gal4 × UAS-S65T-GFP transgenic flies. GFP labels the epithelial glia (eg) surrounding cartridges. There are also visible thin processes (double arrowheads) invading the cartridge. One of the cartridges contains schematic representation of its main components, namely the terminals of R1–R6 photoreceptors (gray outline) and the axons of L1, L2 monopolar cells (white outline). Scale bar: 5 μm. **(C)** The pattern of α5 Mab (Hybridoma) immunolabeling of Na^+^/K^+^-ATPase α-subunit in the cartridges of lamina. The strongest signal comes from the epithelial glia (eg). As in **(B)**, one of the cartridges contains schematic representation of R1–R6 terminals (gray outline) and the axons of L1 and L2 (white outline). Scale bar: 5 μm. **(D)** GFP fluorescence in the lamina glial cells of Repo-Gal4 × UAS-S65T-GFP transgenic flies. Epithelial glia (eg) are localized in the synaptic part of the lamina (Ln). Their cell bodies with nuclei (arrows) are located in the distal part of the neuropil, whereas their processes (arrowheads) reach the proximal part of the neuropil. Lc, lamina cortex; M, medulla. Scale bar: 5 μm. **(E)** Immunoreactivity to the α-subunit of the Na^+^/K^+^-ATPase in the longitudinal section of lamina. Similarly as in the lamina cross section **(C)**, the strongest fluorescence is visible in the membrane of cell bodies (arrows) and processes (arrowheads) of the epithelial glia. Scale bar: 5 μm.

Studies on the housefly, *Musca domestica* and the fruit fly, *Drosophila melanogaster* have shown that in the visual system of Diptera (Figure 1), the circadian plasticity manifests itself both in the retina of the large compound eye (Figure 1A) (Chen et al., 1992) and in the first visual neuropil beneath the compound eye, the lamina (Figure 1B) (Pyza and Górska-Andrzejak, 2008; Pyza, 2010). In the retina, the circadian clock regulates the process of phototransduction, the sensitivity of photoreceptors to light, and their adaptation to changing light conditions (Giebultowicz, 2000; Pyza, 2010). In the underlying lamina, the circadian control is even more pronounced (Pyza and Meinertzhagen, 1997). In the so called cartridges—the synaptic units of lamina neuropil (Figure 2)—both the terminals of photoreceptors (R1–R6) and the axons of their most conspicuous postsynaptic partners (the L1 and L2 interneurons, cf. Figure 2A) exhibit robust structural rhythms (Pyza and Meinertzhagen, 1995, 1997, 1999; Górska-Andrzejak et al., 2005; Barth et al., 2010). It has been shown that in the fruit fly the volume of photoreceptor terminals changes in a circadian manner (Barth et al., 2010), whereas in the housefly the endogenous reorganization of organelles within R1–R6 terminals is maintained under circadian modulation (Pyza and Meinertzhagen, 1997). In *Musca*, the number of screening pigment granules and the number of inter-receptor invaginations from neighboring terminals show circadian changes (Pyza and Meinertzhagen, 1997). The number of synaptic contacts between R1 and R6 terminals and axons of L1, L2 monopolar cells (the tetrad synapses) also undergoes certain changes over the course of 24 h, but this modulation was found to be rather weak and not of circadian origin (Pyza and Meinertzhagen, 1993). In case of *Drosophila*, changes in the number of tetrad presynaptic ribbons have been reported as circadian by Barth et al. (2010). Nevertheless, additional studies that could provide more quantitative insight into the origin of tetrads daily fluctuations would be helpful in clarifying this issue.

Since photoreceptors of the compound eye belong to the peripheral oscillators expressing the core genes of the circadian clock - *period* (*per*) and *timeless* (*tim*) (Siwicki et al., 1988; Zerr et al., 1990; Cheng and Hardin, 1998)—their circadian plasticity might be expected. On the other hand, until now the expression of clock genes have not been detected in their postsynaptic partners—the L1 and L2 monopolar cells. Yet, they display robust circadian rhythms (Pyza and Meinertzhagen, 1993, 1995, 1999; Górska-Andrzejak et al., 2005; Weber et al., 2009). The axons of L1 and L2 in the lamina of three fly species: *Musca domestica, Drosophila melanogaster*, and *Calliphora vicina* (the blow fly) change their size in a circadian manner, though in a different pattern for each species. These patterns are correlated with the increase in fly's daily locomotor activity (Pyza and Meinertzhagen, 1993, 1999; Pyza and Cymborowski, 2001). In the housefly, also the number of so called feedback synapses in L2 axons shows circadian fluctuations (Pyza and Meinertzhagen, 1993). The feedback synapses form back onto the R1–R6 terminals in the proximal lamina (Meinertzhagen and Sorra, 2001; Górska-Andrzejak et al., 2013). Considering that the hyperpolarization of L2 by current injection does not alter the photoreceptors response (Laughlin and Osorio, 1989), the functional significance of transmission at these synapses is not entirely clear and the precise meaning of its circadian nature is difficult to explain. However, the network of interneurons, with L2 among them, does modulate the speed and amplitude of photoreceptors response: when the signal transfer from photoreceptors to interneurons is low, e.g., in dim light condition, the synaptic feedback increases to boost photoreceptors output (Zheng et al., 2006). Taking this into account, one can expect that the L2 monopolar cell at least partly shapes the photoreceptor's response by negative feedback loop, even though it forms only a few feedback synapses (Meinertzhagen and Sorra, 2001). This influence of L2 on photoreceptors output appears to be controlled by the circadian clock because their number increases at the beginning of the night both in LD and DD conditions (Pyza and Meinertzhagen, 1993).

The circadian remodeling of synaptic contacts in Diptera visual system manifests itself also in daily fluctuations in the level of expression of the presynaptic protein Bruchpilot (BRP) (Górska-Andrzejak et al., 2013), which has been shown to localize to the platform of photoreceptor synaptic ribbons (Górska-Andrzejak et al., 2009a; Hamanaka and Meinertzhagen, 2010) and to play the role of the master organizer of the synaptic active zone in *Drosophila* neuromuscular junction (Fouquet et al., 2009). When examined in the distal part of *Drosophila* lamina in LD (12 h of light and 12 h of darkness—12:12), the level of BRP increases twice: at the beginning of the day and at the beginning of the night (Górska-Andrzejak et al., 2013). Interestingly, while the evening peak depends on the circadian input in an essential way, the morning peak of BRP abundance does not appear to be controlled by the circadian clock depending rather on light influence and phototransduction pathway in the retina photoreceptors. Significant alterations in the level of BRP over the course of a 24 h day (Górska-Andrzejak et al., 2013) imply light-dependent and clock-dependent control over the lamina synapses, and ultimately over the neuronal transmission of photic information in the lamina.

**Figure 3:**
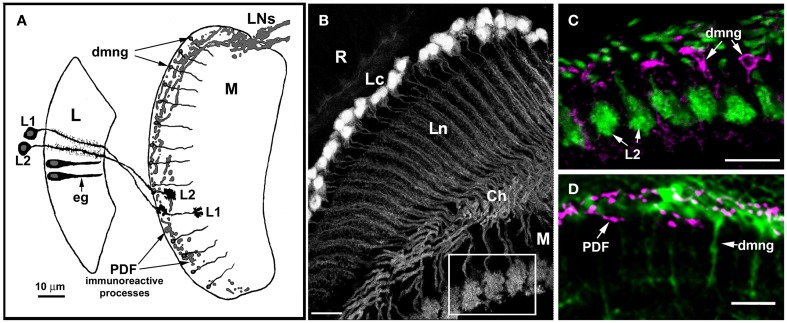
**Anatomical relationships between glial cells (eg, epithelial glial cells of the lamina, dmng, distal medulla neuropil glia) and their putative targets, the L1 and L2 monopolar cells, as well as terminals of some clock neurons (LNs). (A)** Diagram showing the relative locations of L1 and L2 in the lamina and distal medulla, the terminals of PDF immunoreactive lateral neurons (LNs), lamina neuropil epithelial glia (eg), and distal medulla neuropil glia (dmng). L, lamina; M, medulla. **(B)** Morphology of the L2 monopolar cell in the optic lobe of 21D-GAL4 × UAS-S65T-GFP transgenic flies. Cell bodies of L2 are distributed beneath the retina (R), in the region of the lamina cortex (Lc); the axons and dendrites of these cells are in the lamina neuropil (Ln), and their terminals in the medulla (M). Ch, external chiasm. Scale bar: 10 μm. **(C,D)** The region of the medulla seen as an insert in **(B)**. **(C)** GFP labeled terminals of L2 and distal medulla neuropil glia (dmng) showing Ebony-like immunoreactivity. Scale bar: 10 μm. **(D)** The distal medulla neuropil glia (dmng) of Repo-Gal4 × UAS-S65T-GFP transgenic flies and terminals of PDH-immunoreactive LNs (PDF) labeled with anti-PDF antibody. The PDF-positive LNs send projections into the region where the terminals of L2 and Ebony-expressing dmng are located. Scale bar: 10 μm.

Studies on transgenic lines of *Drosophila* (21D-Gal4 × UAS-GFP), in which the morphology of L2 was labeled by cytoplasmic or membranous GFP reporter protein revealed, that in addition to the circadian changes in axon size and the number of feedback synapses, the entire structure of this cell (Figure 3) undergoes daily remodeling (Górska-Andrzejak et al., 2005; Weber et al., 2009). The size of cell nuclei and, more importantly, the size of a dendritic tree in the lamina also oscillate during the 24 h day, with the highest amplitude of changes at the beginning of the day (Górska-Andrzejak et al., 2005; Weber et al., 2009). Interestingly, L2 dendrites that are postsynaptic to photoreceptors are the longest at the beginning of the day (Weber et al., 2009), which coincides with the increase of the number of tetrad synapses in photoreceptor terminals (shown in *Musca*) and with the level of BRP in the lamina (shown in *Drosophila*) (Pyza and Meinertzhagen, 1993; Górska-Andrzejak et al., 2013). This coincidence strongly suggests the possibility of correlation, which however should be confirmed in a direct experiment. Presumably, there are also corresponding changes in L1, which partners L2 (Figures 2A, 3A), although analogous studies of L1 morphology have been so far hindered by the lack of a driver for L1.

The precise mechanism underlying generation of circadian rhythmicity in the monopolar cells of the lamina remains largely unknown. However, if L1 and L2 are not hosting the circadian oscillators themselves, than they must receive the circadian input as the target cells located downstream in the circadian pathway. The increasing amount of data shows that the circadian input to the lamina may originate not only from the retina photoreceptors (Cheng and Hardin, 1998) and the circadian pacemaker neurons of the brain (Bałys and Pyza, 2001; Damulewicz and Pyza, 2011), but also from glial cells of the optic lobe (Pyza and Górska-Andrzejak, 2004; Górska-Andrzejak et al., 2009b, 2013).

Glial cells of the *Drosophila* optic lobe (Figure 1C) belong to surface, cortex, neuropil, and tract glia (Edwards and Meinertzhagen, 2010). They differ by their location, morphology, gene expression, and function (recently described in Edwards and Meinertzhagen, 2010; Hartenstein, 2011; Edwards et al., 2012). Underneath the retina of the large compound eye, at the surface of the lamina, there are two distinct subtypes of the surface glia: the fenestrated glia (fg; perineurial layer) and the pseudocartridge glia (psdg; subperineurial layer). Below them, the lamina is populated by two types of the cortex glia (also called “satellite,” when located in the visual system) and two types of the neuropil glia. The so called distal (dsg) and proximal (psg) satellite glia ensheath the cell bodies of monopolar neurons and the photoreceptor axon bundles in the lamina cortex, whereas the epithelial (eg) and marginal (mg) glia reside in the lamina synaptic neuropil, where they ensheath neuronal processes (Figure 1C). The epithelial glia has been recently reported to be the lamina counterpart of the so called astrocyte-like neuropil glia, even though its columnar morphology (Figure 2D) does not resemble the astrocyte-like one (Edwards et al., 2012). The cortex glia and the neuropil glia are also present in the second visual neuropil or medulla. Medulla cortex glia (mcg) typically for this type of glia form a mesh of processes that encapsulate the neighboring neuronal cell bodies, whereas the medulla neuropil glia extend their processes deep into the synaptic neuropil (Figure 1C). Distinct types of the medulla neuropil glia have been recently described based on differences in morphology and localization (Edwards et al., 2012). There are at least two morphologically different types of the neuropil glia in the distal part of the medulla neuropil (dmng), with the first one representing the astrocyte-like glia (aslg) and the second having different kind of morphology (ng) (Figure 1C). Apart from the distal part, the astrocyte-like glia reside in other parts of this neuropil as well; the serpentine glia (spg) in the posterior margin of M7 (the serpentine layer) and the chandelier glia (chg) at the base of the medulla (Edwards et al., 2012). Like the first two, the third visual neuropil—the lobula complex is populated by cortex and neuropil glia as well (Eule et al., 1995; Tix et al., 1997). The astrocyte-like glia have also been reported among the lobula neuropil glia, although not characterized further (Edwards et al., 2012). The axon tracts projecting between the visual neuropils are wrapped by the tract glia (Figure 1C) (Edwards and Meinertzhagen, 2010). These are glia of the outer (ocg) and the inner (icg) optic chiasmata (between the lamina and the medulla and between the medulla and the lobula, respectively) (Figure 1C). When the outer chiasm is populated by the giant and the small ocg, the inner chiasm hosts only the giant glia. Each of the above mentioned types/subtypes of glia not only has a unique morphology and function, but also contacts a unique group of optic lobe neurons (Edwards et al., 2012).

## Glial oscillators

Glial cells of Diptera seem capable of taking part in the circadian modulation of neuronal circuitry of the lamina, first and foremost because they themselves contain circadian oscillators (Zeng et al., 1994; Jackson, 2011). It has been known for more than two decades that certain types of glial cells express *per*, the core gene of the mechanism for keeping circadian time, and that the abundance of its product (PER) in glia, like in the neurons, fluctuates in a circadian manner (Siwicki et al., 1988; Zerr et al., 1990; Ewer et al., 1992). The fruit fly glia express also another clock gene: *tim* (Suh and Jackson, 2007). In the transcriptional negative feedback loop of *Drosophila* molecular clock (the core loop), PER and TIM proteins form heterodimers to enter the nucleus and inhibit the transcription of their own genes by repressing transcription activator composed of Clock (CLK) and Cycle (CYC). This generates oscillations in the level of their expression in the cell (Hardin, 2005). If not all, than at least those glial cells that contain the PER-based molecular oscillator are well equipped to function as rhythm generators and modulators (Zeng et al., 1994). In *Drosophila*, the glial cells of the visual system also belong to the *per* expressing glia (Siwicki et al., 1988; Ewer et al., 1992).

## Glia derived circadian modulation of the lamina neuronal circuitry

### Involvement of the epithelial glia in the circadian volume changes of l1, l2 axons

In the lamina synaptic neuropil of *Musca domestica*, the spontaneous cyclical changes that follow a circadian rhythm have been detected not only in the morphology of the L1 and L2 monopolar cells, but also in morphology of the epithelial glia (Figure 2D) (Pyza and Górska-Andrzejak, 2004). In *Musca* and *Drosophila* three of such cells surround each synaptic cartridge (Boschek, 1971; Meinertzhagen and O'Neil, 1991) sending their projections toward its inside (Figures 2A,B). Squeezing between much bigger profiles of R1–R6 and L1, L2, the glial projections localize themselves in close proximity to the neuronal profiles and their synaptic connections. In case of photoreceptors, the epithelial glia make characteristic close invaginating appositions into their terminals (Figure 2A): the so called capitate projections (Edwards and Meinertzhagen, 2010). The precise function of these intimate connections between photoreceptors and glial cells is still quite obscure. They are known, however, to be involved in vesicle endocytosis (Fabian-Fine et al., 2003).

The epithelial glia have been found to undergo daily alterations in cell morphology (Pyza and Górska-Andrzejak, 2004). It is not much of a surprise in view of close association of the lamina epithelial glia with the photoreceptor terminals and axons of L1, L2 monopolar cells that display circadian changes. Epithelial glial cells of *Musca* expand at night and shrink during the day, exactly in antiphase to daily changes of L1 and L2 neurons (Pyza and Górska-Andrzejak, 2004). (Axons of L1 and L2 swell during the day, and shrink by night). This suggests some kind of a compensatory relationship between L1, L2 and surrounding epithelial glia. Possibly, glial cells that contain the PER-based oscillator (epithelial glia express *per*; Siwicki et al., 1988) as the part of the circadian system provide some degree of control over the volume changes in L1, L2. The possibility of such control is corroborated by data showing that daily changes in glia morphology influence the rhythmic structural changes of L1 and L2 axons (Pyza and Górska-Andrzejak, 2004). Disrupting the metabolism of glia by injecting glial metabolic toxins, e.g., fluorocitrate or iodoacetate (Kitano et al., 2003) affects the neuronal rhythm by increasing its amplitude (Pyza and Górska-Andrzejak, 2004). Alterations in the neuronal rhythm occur also when the communication between the lamina cells via the gap junction channels is disrupted (Pyza and Górska-Andrzejak, 2004). Most of the gap junctions in the lamina are formed between glial cells (Saint Marie et al., 1984). Therefore, the uncoupling effect of the application of gap junction closing agent, such as octanol (Pappas et al., 1996), is observed predominantly on the “glial part” of the lamina. The contribution of gap junctions formed between neurons, however, cannot be entirely ruled out. In the condition of decreased coupling between glial cells, the amplitude of both glial and neuronal rhythm was observed to be noticeably smaller (Pyza and Górska-Andrzejak, 2004). In fact, in case of L2, the differences between the volume of day-axons and night-axons were no longer significant. Such results indicate, that the epithelial glia of the lamina modulate the circadian rhythmicity of the target neurons L1 and L2. Interestingly, the decreasing cell volume effect of octanol in neurons was stronger during the day, whereas in glia it was stronger during the night (Pyza and Górska-Andrzejak, 2004).

### Circadian expression of Na^+^/K^+^-ATPase in the visual system

In *Drosophila melanogaster*, the circadian changes in the volume of lamina monopolar cells are accompanied by daily changes in the expression of the Na^+^/K^+^-ATPase, the sodium pump (Górska-Andrzejak et al., 2009b). The sodium pump is the major pump that regulates the ionic homeostasis in many different cell types (Lingrel et al., 1990). Therefore, its engagement in the mechanism of neuronal and glial cells volume regulation, including diurnal modulation, might have been expected. In *Drosophila* optic lobe, the expression of Na^+^/K^+^-ATPase were indeed observed to be under the circadian control at both mRNA and protein level (Górska-Andrzejak et al., 2009b). However, of the α and β subunits of the sodium pump (responsible for the catalytic and regulatory functions, respectively), only the first one showed a robust, *per*-dependent circadian rhythm of concentration (Górska-Andrzejak et al., 2009b).

The relative amount of the α subunit protein was at the highest level at the beginning of the night and at the lowest level at the end of the night, as revealed by the immunolabeling intensity for this subunit (Górska-Andrzejak et al., 2009b). Such pattern of daily changes of the sodium pump coincides with the abovementioned pattern of oscillations in L1 and L2 axon sizes (Pyza and Meinertzhagen, 1999). It is also interesting to note that the immunospecific labeling for the α subunit was particularly strong in the first and the second optic neuropil. It was detected in most cells of the optic lobe, but in the lamina the particularly intense signal was visible in the epithelial glia (Figures 2C,E), especially in their membranes, where the protein is active (Figure 2E) (Górska-Andrzejak et al., 2009b). It is thus possible that predominantly the glial oscillators contribute to the rhythm of the sodium pump. In view of these observations, L1 and L2 appear as likely targets for circadian modulation driven by epithelial glia, which at certain times of the day might provide such ion buffering capacity that is required by axons of highly active neurons.

It was also reported that the Na^+^/K^+^-ATPase may have a pump-independent function. It is required for cell junctions formation (Genova and Fehon, 2003; Paul et al., 2007). Therefore, it is possible that Na^+^/K^+^-ATPase is involved in neuron—glia communication. If this were the case, the observed circadian changes in its expression in the optic lobe of *Drosophila* would additionally mean the existence of the circadian control over neuron-glia communication.

In case of the β-subunit of the Na^+^/K^+^-ATPase (encoded by the *Nervana 2*), the results were less conclusive (Górska-Andrzejak et al., 2009b). The fluorescence intensity of GFP reporter revealed only small, statistically insignificant differences between day and night. However, it might have been partly due to limitations imposed by the variant of the GFP reporter (S65T-GFP) driven by *Nrv2* (Sun et al., 1999) used in these studies. Interestingly, the GFP signal was strong in the glial cells of the medulla, especially in the distal medulla neuropil glia (dmng) (Figures 1C, 3). Their cell bodies are located between the cortex and the neuropil of the medulla, whereas their long processes penetrate the neuropil. It is difficult to determine to which type of the distal medulla glia (aslg or ng, perhaps both) the *Nerv2*-expressing glia belong to. Still, their processes run close to the terminals of L2 and L1 (Figures 3A,C), and close to dense varicose arborization of the terminals of the circadian clock neurons—the lateral neurons (LNs) (Figures 3A,D). LNs terminals are immunoreactive to pigment dispersing factor (PDF), a transmitter in the circadian system (Saifullah and Tomioka, 2003) and synchronizer of different clock neurons in the system (Lin et al., 2004). Apart from close localization to LNs terminals that release the PDF, these glial cells express *per*. Because of such characteristics, these type of glia may have great capacity to modulate the circadian rhythmicity in neurons, such as L1 or L2. However, further studies are needed to establish their role in this process.

### Glial influence on circadian pattern of BRP expression

Recent findings also suggest that glial cells influence the above described pattern of circadian expression of the presynaptic BRP in distal lamina of *D. melanogaster*. It appears that the bimodal pattern of daily changes in the amount of BRP in the cartridges of lamina depends not only on circadian oscillators in photoreceptors and the clock neurons of the brain but also on the glial oscillators (Górska-Andrzejak et al., 2013).

In *per*^0^ mutants, in which the circadian system is disrupted, both peaks in BRP abundance disappear and the rhythm is abolished. However, when only the glial circadian oscillator is disrupted (by dominant negative form of the Cycle protein, CycΔ#103) or the expression of *per* is silenced exclusively in the glia (by *per-RNAi* construct), the daily changes in BRP accumulation in the lamina cartridges still take place, but their pattern is considerably altered (Górska-Andrzejak et al., 2013). Glial cells as the component of the circadian system apparently modulate the pattern of circadian expression of BRP, and therefore they appear to have an influence on daily changes in neurotransmission in the lamina of Diptera.

### Circadian expression of glia-specific protein *ebony*

Studies on the expression and function of *ebony* gene in *Drosophila*, revealed another interesting information about “circadian capacity” of glial cells: they can drive the behavioral circadian rhythms (Suh and Jackson, 2007). *ebony*—the key player in this process—encodes a protein of N-β-alanyl-biogenic amine synthase activity (Ebony), an enzyme that conjugates β-alanine to biogenic amines, such as dopamine and serotonin (Hovemann et al., 1998; Richardt et al., 2003). The fact that *ebony* mutants display arrhythmic locomotor activity (Newby and Jackson, 1991) and that in the head of a non-mutant fly the *ebony* mRNA shows robust circadian cycling (Ueda et al., 2002) strongly suggest that this protein modulates circadian behavior.

The most important, however, is that in *Drosophila* head Ebony localizes exclusively to glial cells. By discovering this fact and showing that glial expression of Ebony rescues the phenotype of *ebony* mutants, Suh and Jackson (2007) demonstrated a connection between glial cells and the circadian control of a locomotor behavior of the fruit fly. They reported Ebony as the first identified glial factor required for behavioral rhythmicity. They also proposed a model in which Ebony participates in the circadian control of dopaminergic functions and circadian activity rhythms (Suh and Jackson, 2007).

In the visual system of *Drosophila*, similarly as in the whole brain, Ebony protein localizes exclusively to glial cells. As Suh and Jackson (2007) reported, the majority of Ebony-positive glia in fly brain belongs to neuropil glia. In the visual system, the Ebony-positive glia have been identified as the lamina epithelial glia (eg), the neuropil glia of distal medulla (dmng), (Figures 1C, 3C) (Richardt et al., 2003) and recently also the serpentine glia (Figure 1C) (Edwards et al., 2012). These are incidentally the same glial cells that express PER protein (Siwicki et al., 1988; Zerr et al., 1990; Ewer et al., 1992), display circadian changes in morphology and influence circadian rhythms of L1 and L2 (eg) (Pyza and Górska-Andrzejak, 2004), express the Na^+^/K^+^-ATPase in a circadian manner (eg and dmng), and are localized in the area of PDF release from the terminals of LNs (dmng) (Górska-Andrzejak et al., 2009b). Ebony-positive glia of the visual system are therefore definitely involved in its circadian modulation.

In the visual system of *Drosophila* Ebony is required for the high level of daytime activity. It conjugates β-alanine to histamine forming β-alanyl-histamine, or carcinine (Borycz et al., 2002; Richardt et al., 2003; Ziegler et al., 2012). By expressing Ebony, epithelial glial cells take part in the recycling of the photoreceptors neurotransmitter—histamine, and appear to regulate circadian photoreception (extensively reviewed in Edwards and Meinertzhagen, 2010 and Jackson, 2011).

## Conclusions

The visual system of Diptera is well established as a model for the circadian rhythms studies. The photoreceptor cells of the large compound eye of *Drosophila* express clock genes and are by themselves the peripheral oscillators. The optic lobe of *Drosophila*, on the other hand, contains the circadian system that consists of two components: the circadian clock neurons—the LNs and the PER-expressing glial cells. The main directions of research have focused on different groups of clock neurons, their circuitry, and ways of transmitting the circadian information—circadian neurotransmitters and their receptors. In comparison with the clock neurons, the glial cells have received considerably less attention.

Incidentally, certain types of glial cells may actually be called “glial clocks” (Jackson, 2011). They contain PER-based circadian oscillators (Siwicki et al., 1988; Zerr et al., 1990; Ewer et al., 1992; Zeng et al., 1994; Jackson, 2011) and appear to be the integral part of the circadian system as partners for the clock neurons rather than only supportive cells (Pyza and Górska-Andrzejak, 2004; Suh and Jackson, 2007; Górska-Andrzejak et al., 2009b, 2013; Jackson, 2011; Ng et al., 2011).

Out of the four main glial types described in the brain and visual system of *Drosophila* (Edwards and Meinertzhagen, 2010), only certain types of the neuropil glia have been shown to take part in circadian modulation of neuronal circuitry (and behavior) (Jackson, 2011). They extend their membranes around axons or axon bundles (Edwards and Meinertzhagen, 2010) and form intimate connections with neurons in the synaptic part (neuropil) of the nervous structure. In the optic lobe of *Drosophila melanogaster* and *Musca domestica* these are the epithelial glial cells of the lamina neuropil and the neuropil glia of the medulla. Both of them modulate the circadian rhythmicity of L1 and L2 interneurons, which are the output cells in the circadian system of the optic lobe (Figure 3) (Pyza and Górska-Andrzejak, 2004; Górska-Andrzejak et al., 2009b). The precise function of the medulla neuropil glia in modulating L1, L2 rhythms, however, has been described mainly through indirect evidence and requires further studies.

Obviously, the clock neurons (LNs) play a crucial role in *Drosophila* pacemaker, and in the circadian plasticity of the visual system. Breaking the connection between the housefly's optic lobe and the rest of the brain abolishes the shrinking and swelling rhythm of L1, L2 axons changes (Bałys and Pyza, 2001). The recent studies revealed also the direct input from LNs to the lamina, which uses an ITP-like peptide as a neurotransmitter (Damulewicz and Pyza, 2011).

One might wonder then why would clock neurons need glia to convey the circadian information if they have means to directly send the signal to the target cells, like L1, L2? A good answer may have been given in the studies on glia involvement in circadian axon changes in L1 and L2 (Pyza and Górska-Andrzejak, 2004). Pathologic glia with disrupted metabolism are no longer able to counteract the neuronal size changes, which results in excessive increase in the amplitude of the rhythm (Pyza and Górska-Andrzejak, 2004). This suggest not only that L1 and L2 are the proximal targets for glial control of their circadian plasticity but also that the function of these glial cells is to stabilize the proper level of daily changes. To reach these goals the glial cells appear to work as a synchronized network. Diminishing the normal level of inter-glial/glial-neuronal synchronization by closing gap junction channels between them results in flattening of the neuronal rhythm (Pyza and Górska-Andrzejak, 2004). Localization and strong expression of PER in the medulla neuropil glia, which contact the PDF immunoreactive terminals of LNs (Figure 3C) implies that these cells may receive instructive circadian signals from clock neurons. The glial clocks, however, are also capable of modulating the clock neurons: the recent studies on *Drosophila* neuropil glia of astrocyte type showed that the glial cells can physiologically modulate the circadian clock neurons (Ng et al., 2011). It has also been shown that the activity of the two ubiquitous transcription factor families that are involved in numerous cellular processes: cAMP response element-binding protein (CREB) and nuclear factor kappa-B (NF-κB), remains under circadian regulation both in neurons and in glia (Tanenhaus et al., 2012). In view of the observations presented in this short review one may conclude that glial clocks and neuronal clocks are partners.

### Conflict of interest statement

The author declares that the research was conducted in the absence of any commercial or financial relationships that could be construed as a potential conflict of interest.

## References

[B1] Bałys M. Pyza E. (2001). Localization of the clock controlling circadian rhythms in the first neuropile of the optic lobe in the housefly. J. Exp. Biol. 204, 3303–3310 11606604 10.1242/jeb.204.19.3303

[B2] Barth M. Schultze M. Schuster C. M. Strauss R. (2010). Circadian plasticity in photoreceptor cells controls visual coding efficiency in *Drosophila melanogaster*. PLoS ONE 5:e9217 10.1371/journal.pone.0009217 20169158 PMC2821403

[B2a] Borycz J. Borycz J. A. Loubani M. Meinertzhagen I. A. (2002). *tan* and *ebony* genes regulate a novel pathway for transmitter metabolism at fly photoreceptor terminals. J. Neurosci. 22, 10549–10557 12486147 10.1523/JNEUROSCI.22-24-10549.2002 PMC6758454

[B3] Boschek C. B. (1971). On the fine structure of the peripheral retina and lamina ganglionaris of the fly, *Musca domestica*. Z. Zellforsch. Mikrosk. Anat. 118, 369–409 5566322 10.1007/BF00331193

[B4] Chen D. M. Christianso J. S. Sapp R. J. Stark W. S. (1992). Visual receptor cycle in normal and period mutant *Drosophila*: microspectrophotometry, electrophysiology, and ultrastructural morphometry. Vis. Neurosci. 9, 125–135 1504021 10.1017/s0952523800009585

[B5] Cheng Y. Hardin P. E. (1998). *Drosophila* photoreceptors contain an autonomous circadian oscillator that can function without period mRNA cycling. J. Neurosci. 18, 741–750 9425016 10.1523/JNEUROSCI.18-02-00741.1998 PMC6792536

[B6] Damulewicz M. Pyza E. (2011). The clock input to the first optic neuropil of *Drosophila melanogaster* expressing neuronal circadian plasticity. PLoS ONE 6:e21258 10.1371/journal.pone.0021258 21760878 PMC3124489

[B7] Edwards T. N. Meinertzhagen I. A. (2010). The functional organisation of glia in the adult brain of *Drosophila* and other insects. Prog. Neurobiol. 90, 471–497 10.1016/j.pneurobio.2010.01.001 20109517 PMC2847375

[B8] Edwards T. N. Nuschke A. C. Nern A. Meinertzhagen I. A. (2012). Organization and metamorphosis of glia in the *Drosophila* visual system. J. Comp. Neurol. 520, 2067–2085 10.1002/cne.23071 22351615

[B8a] Eule E. Tix S. Fischbach K. F. (1995). Glial cells in the optic lobe of *Drosophila melanogaster*. Flybrain poster. Available online at: http://www.flybrain.org/Flybrain/html/poster/ (Accession no. PP 00004).

[B9] Ewer J. Frisch B. Hamblen-Coyle M. J. Rosbash M. Hall J. C. (1992). Expression of the *period* clock gene within different cell types in the brain of *Drosophila* adults and mosaic analysis of these cells' influence on circadian behavioral rhythms. J. Neurosci. 12, 3321–3349 1382123 10.1523/JNEUROSCI.12-09-03321.1992 PMC6575742

[B10] Fabian-Fine R. Verstreken P. Hiesinger P. R. Horne J. A. Kostyleva R. Zhou Y. (2003). Endophilin promotes a late step in endocytosis at glial invaginations in *Drosophila* photoreceptor terminals. J. Neurosci. 23, 10732–10744 14627659 10.1523/JNEUROSCI.23-33-10732.2003 PMC6740933

[B11] Fouquet E. Owald D. Wichmann C. Mertel S. Depner H. Dyba M. (2009). Maturation of active zone assembly by *Drosophila* Bruchpilot. J. Cell Biol. 186, 129–145 10.1083/jcb.200812150 19596851 PMC2712991

[B12] Frenkel L. Ceriani M. F. (2011). Circadian plasticity: from structure to behavior. Int. Rev. Neurobiol. 99, 107–138 10.1016/B978-0-12-387003-2.00005-7 21906538

[B13] Genova J. L. Fehon R. G. (2003). Neuroglian, Gliotactin, and the Na^+^/K^+^ ATPase are essential for septate junction function in *Drosophila*. J. Cell Biol. 161, 979–989 10.1083/jcb.200212054 12782686 PMC2172966

[B14] Giebultowicz J. M. (2000). Molecular mechanism and cellular distribution of insect circadian clocks. Annu. Rev. Entomol. 45, 769–793 10.1146/annurev.ento.45.1.769 10761596

[B15] Górska-Andrzejak J. Keller A. Raabe T. Kilianek L. Pyza E. (2005). Structural daily rhythms in GFP-labelled neurons in the visual system of *Drosophila melanogaster*. Photochem. Photobiol. Sci. 4, 721–726 10.1039/b417023g 16121283

[B16] Górska-Andrzejak J. Makuch R. Stefan J. Görlich A. Semik D. Pyza E. (2013). Circadian expression of the presynaptic active zone protein Bruchpilot in the lamina of *Drosophila melanogaster*. Dev. Neurobiol. 73, 14–26 10.1002/dneu.22032 22589214

[B17] Górska-Andrzejak J. Nianko E. Pyza E. (2009a). Circadian expression of the presynaptic active zone protein Bruchpilot in the lamina of *Drosophila melanogaster*. J. Neurogenet. 23, S38–S39 Meeting Abstract: V21. 10.1002/dneu.22032 22589214

[B18] Górska-Andrzejak J. Salvaterra P. M. Meinertzhagen I. A. Krzeptowski W. Görlich A. Pyza E. (2009b). Cyclical expression of Na^+^/K^+^-ATPase in the visual system of *Drosophila melanogaster*. J. Insect Physiol. 55, 459–468 10.1016/j.jinsphys.2009.02.003 19428365 PMC2721802

[B19] Hamanaka Y. Meinertzhagen I. A. (2010). Immunocytochemical localization of synaptic proteins to photoreceptor synapses of *Drosophila melanogaster*. J. Comp. Neurol. 518, 1133–1155 10.1002/cne.22268 20127822 PMC4029604

[B20] Hardin P. E. (2005). The circadian timekeeping system of *Drosophila*. Curr. Biol. 15, 714–722 10.1016/j.cub.2005.08.019 16139204

[B21] Hartenstein V. (2011). Morphological diversity and development of glia in *Drosophila*. Glia 59, 1237–1252 10.1002/glia.21162 21438012 PMC3950653

[B22] Hovemann B. T. Ryseck R. P. Walldorf U. Störtkuhl K. F. Dietzel I. D. Dessen E. (1998). The *Drosophila* ebony gene is closely related to microbial peptide synthetases and shows specific cuticle and nervous system expression. Gene 221, 1–9 10.1016/S0378-1119(98)00440-5 9852943

[B23] Jackson F. R. (2011). Glial cell modulation of circadian rhythms. Glia 59, 1341–1350 10.1002/glia.21097 21732426 PMC3156034

[B24] Kitano T. Nisimaru N. Shibata E. Iwasaka H. Noguchi T. Yokoi I. (2003). Monocarboxylates and glucose utilization as energy substrates in rat brain slices under selective glial poisoning–a 31P NMR study. Mol. Cell. Biochem. 244, 77–81 12701813

[B25] Laughlin S. B. Osorio D. (1989). Mechanisms for neural signal enhancement in the blowfly compound eye. J. Exp. Biol. 144, 113–146

[B26] Lin Y. Stormo G. D. Taghert P. H. (2004). The neuropeptide pigment-dispersing factor coordinates pacemaker interactions in the *Drosophila* circadian system. J. Neurosci. 24, 7951–7957 10.1523/JNEUROSCI.2370-04.2004 15356209 PMC6729918

[B27] Lingrel J. B. Orlowski J. Shull M. M. Price E. M. (1990). Molecular genetics of Na, K-ATPase. Prog. Nucleic Acid Res. Mol. Biol. 38, 37–89 2158121 10.1016/s0079-6603(08)60708-4

[B28] Mehnert K. I. Cantera R. (2011). Circadian rhythms in the morphology of neurons in *Drosophila*. Cell Tissue Res. 344, 381–389 10.1007/s00441-011-1174-x 21562943

[B29] Meinertzhagen I. A. O'Neil S. D. (1991). Synaptic organization of columnar elements in the lamina of the wild type in *Drosophila melanogaster*. J. Comp. Neurol. 305, 232–263 10.1002/cne.903050206 1902848

[B30] Meinertzhagen I. A. Sorra K. E. (2001). Synaptic organization in the fly's optic lamina: few cells, many synapses and divergent microcircuits. Prog. Brain Res. 131, 53–69 11420968 10.1016/s0079-6123(01)31007-5

[B31] Newby L. M. Jackson F. R. (1991). *Drosophila ebony* mutants have altered circadian activity rhythms but normal eclosion rhythms. J. Neurogenet. 7, 85–101 1903161 10.3109/01677069109066213

[B32] Ng F. S. Tangredi M. M. Jackson F. R. (2011). Glial cells physiologically modulate clock neurons and circadian behavior in a calcium-dependent manner. Curr. Biol. 21, 625–634 10.1016/j.cub.2011.03.027 21497088 PMC3081987

[B33] Pappas C. A. Rioult M. G. Ransom B. R. (1996). Octanol, a gap junction uncoupling agent, changes intracellular [H+] in rat astrocytes. Glia 16, 7–15 10.1002/(SICI)1098-1136(199601)16:1<7::AID-GLIA2>3.0.CO;2-2 8787769

[B34] Paul S. M. Palladino M. J. Beitel G. J. (2007). A pump-independent function of the Na, K-ATPase is required for epithelial junction function and tracheal tube-size control. Development 134, 147–155 10.1242/dev.02710 17164420 PMC1955469

[B35] Pyza E. (2010). Circadian rhythms in the fly's visual systems, in Encyclopedia of the Eye, Vol. 1, ed Darlene A. D. (Oxford: Academic Press), 302–311

[B37] Pyza E. Cymborowski B. (2001). Circadian rhythms in behaviour and in the visual system of the blowfly *Calliphora vicina*. J. Insect Physiol. 47, 897–904

[B38] Pyza E. Górska-Andrzejak J. (2004). Involvement of glial cells in rhythmic size changes in neurons of the housefly's visual system. J. Neurobiol. 59, 205–215 10.1002/neu.10307 15085538

[B39] Pyza E. Górska-Andrzejak J. (2008). External and internal inputs affecting plasticity of dendrites and axons of the fly's neurons. Acta Neurobiol. Exp. (Wars) 68, 322–333 18511964 10.55782/ane-2008-1698

[B40] Pyza E. Meinertzhagen I. A. (1993). Daily and circadian rhythms of synaptic frequency in the first visual neuropile of the housefly's (*Musca domestica* L.) optic lobe. Proc. Biol. Sci. 254, 97–105 10.1098/rspb.1993.0133 8290615

[B41] Pyza E. Meinertzhagen I. A. (1995). Monopolar cell axons in the first optic neuropil of the housefly, *Musca domestica* L., undergo daily fluctuations in diameter that have a circadian basis. J. Neurosci. 15, 407–418 7823145 10.1523/JNEUROSCI.15-01-00407.1995 PMC6578271

[B42] Pyza E. Meinertzhagen I. A. (1997). Circadian rhythms in screening pigment and invaginating organelles in photoreceptor terminals of the housefly's first optic neuropile. J. Neurobiol. 32, 517–529 10.1002/(SICI)1097-4695(199705)32:5<517::AID-NEU6>3.0.CO;2-8 9110262

[B43] Pyza E. Meinertzhagen I. A. (1999). Daily rhythmic changes of cell size and shape in the first optic neuropil in *Drosophila melanogaster*. J. Neurobiol. 40, 77–88 10.1002/(SICI)1097-4695(199907)40:1<77::AID-NEU7>3.0.CO;2-0 10398073

[B44] Richardt A. Kemme T. Wagner S. Schwarzer D. Marahiel M. A. Hovemann B. T. (2003). Ebony, a novel nonribosomal peptide synthetase for beta-alanine conjugation with biogenic amines in *Drosophila*. J. Biol. Chem. 278, 41160–41166 10.1074/jbc.M304303200 12900414

[B45] Saifullah A. S. Tomioka K. (2003). Pigment-dispersing factor sets the night state of the medulla bilateral neurons in the optic lobe of the cricket, *Gryllus bimaculatus*. J. Insect Physiol. 49, 231–239 10.1016/S0022-1910(02)00270-6 12769998

[B46] Saint Marie R. L. Carlson S. D. Chi C. (1984). The glial cells of insects, in Insect Ultrastructure, Vol. 2, eds King R. C. Akai H. (New York, NY: Plenum), 435–475

[B47] Siwicki K. K. Eastman C. Petersen G. Rosbash M. Hall J. C. (1988). Antibodies to the *period* gene product of *Drosophila* reveal diverse tissue distribution and rhythmic changes in the visual system. Neuron 1, 141–150 10.1016/0896-6273(88)90198-5 3152288

[B48] Suh J. Jackson F. R. (2007). *Drosophila* ebony activity is required in glia for the circadian regulation of locomotor activity. Neuron 55, 435–447 10.1016/j.neuron.2007.06.038 17678856 PMC2034310

[B49] Sun B. Xu P. Salvaterra P. M. (1999). Dynamic visualization of nervous system in live *Drosophila*. Proc. Natl. Acad. Sci. U.S.A. 96, 10438–10443 10.1073/pnas.96.18.10438 10468627 PMC17907

[B50] Tanenhaus A. K. Zhang J. Yin J. C. (2012). *In vivo* circadian oscillation of dCREB2 and NF-κB activity in the *Drosophila* nervous system. PLoS ONE 7:e45130 10.1371/journal.pone.0045130 23077489 PMC3471920

[B50a] Tix S. Eule E. Fischbach K. F. Benzer S. (1997). Glia in the chiasms and medulla of the *Drosophila melanogaster* optic lobes. Cell Tissue Res. 289, 397–409 10.1007/s004410050886 9232819

[B51] Ueda H. R. Matsumoto A. Kawamura M. Iino M. Tanimura T. Hashimoto S. (2002). Genome-wide transcriptional orchestration of circadian rhythms in *Drosophila*. J. Biol. Chem. 277, 14048–14052 10.1074/jbc.C100765200 11854264

[B52] Weber P. Kula-Eversole E. Pyza E. (2009). Circadian control of dendrite morphology in the visual system of *Drosophila melanogaster*. PLoS ONE 4:e4290 10.1371/journal.pone.0004290 19173003 PMC2628732

[B53] Zeng H. Hardin P. E. Rosbash M. (1994). Constitutive overexpression of the *Drosophila period* protein inhibits period mRNA cycling. EMBO J. 13, 3590–3598 8062834 10.1002/j.1460-2075.1994.tb06666.x PMC395264

[B54] Zerr D. M. Hall J. C. Rosbash M. Siwicki K. K. (1990). Circadian fluctuations of *period* protein immunoreactivity in the CNS and the visual system of *Drosophila*. J. Neurosci. 10, 2749–2762 2117644 10.1523/JNEUROSCI.10-08-02749.1990 PMC6570283

[B55] Zheng L. de Polavieja G. G. Wolfram V. Asyali M. H. Hardie R. C. Juusola M. (2006). Feedback network controls photoreceptor output at the layer of first visual synapses in *Drosophila*. J. Gen. Physiol. 127, 495–510 10.1085/jgp.200509470 16636201 PMC2151524

[B56] Ziegler A. B. Brüsselbach F. Hovemann B. T. (2012). Activity and co-expression of Drosophila Black with Ebony in fly optic lobes reveals putative cooperative tasks in vision that evade ERG detection. J. Comp. Neurol. 521, 1207–1224 10.1002/cne.23247 23124681

